# Prevalence, concordance and associations of chronic kidney disease by five estimators in South Africa

**DOI:** 10.1186/s12882-020-02018-x

**Published:** 2020-08-27

**Authors:** Nasheeta Peer, Jaya George, Carl Lombard, Krisela Steyn, Naomi Levitt, Andre-Pascal Kengne

**Affiliations:** 1 Non-communicable Diseases Research Unit, South African Medical Research Council, Durban and Cape Town, South Africa; 2 Department of Medicine, University of Cape Town, Cape Town, South Africa; 3 Department of Chemical Pathology, University of the Witwatersrand, and National Health Laboratory Services, Johannesburg, South Africa; 4 Biostatistics Unit, South African Medical Research Council, Cape Town, South Africa; 5 Chronic Disease Initiative for Africa, Department of Medicine, University of Cape Town, Cape Town, South Africa

**Keywords:** Chronic kidney disease, CKD-EPI, Cockcroft-Gault, Cystatin C, MDRD, South Africa

## Abstract

**Background:**

To determine the prevalence, distribution, concordance and associations of chronic kidney disease (CKD) determined by five glomerular filtration rate (GFR) formulae in urban black residents of Cape Town.

**Methods:**

Data collection in this cross-sectional study included interviews, clinical measurements and biochemical analyses, including serum creatinine and cystatin C levels. GFR was based on the CKD Epidemiology Collaboration (CKD-EPI) equations (CKD-EPI creatinine (CKD-EPIcr), CKD-EPI cystatin C (CKD-EPIcys), CKD-EPI creatinine-cystatins (CKD-EPIcr-cys)), Modification of Diet in Renal Disease (MDRD) and Cockcroft-Gault formula (CGF). GFR < 60 mL/min/1.73 m^2^ defined CKD.

**Results:**

Among 392 men and 700 women, mean GFR, was between 114.0 (CKD-EPIcr) and 135.4 mL/min/1.73 m^2^ (CGF) in men, and between 107.5 (CKD-EPIcr-cys) and 173.4 mL/min/1.73 m^2^ (CGF) in women. CKD prevalence ranged from 2.3% (CKD-EPIcr and MDRD) to 5.1% (CKD-EPIcys) in men and 1.6% (CGF) to 6.7% (CKD-EPIcr-cys) in women. The kappa statistic was high between CKD-EPIcr and MDRD (0.934), and CKD-EPIcys and CKD-EPIcr-cys (0.815), but fair-to-moderate between the other eqs. (0.353–0.565). In the basic regressions, older age and body mass index ≥30 kg/m^2^, but not gender, were significantly associated with CKD-EPIcr-defined CKD. In the presence of these three variables, hypertension, heart rate ≥ 90 beats/minute, diabetes and low-density lipoprotein cholesterol were significant predictors of prevalent CKD.

**Conclusions:**

Varying CKD prevalence estimates, because of different GFR equations used, underscores the need to improve accuracy of CKD diagnoses. Furthermore, screening for CKD should be incorporated into the routine assessment of high-risk patients such as those with hypertension or diabetes.

## Background

There has been a global increase in chronic kidney disease (CKD) over the last 2–3 decades [1]; the estimated prevalence is > 10% [2]. Contributing to the rise in CKD is the worldwide increase in prevalence of hypertension, diabetes, obesity and the metabolic syndrome. While these conditions are risk factors for cardiovascular disease (CVD), the presence of CKD further raises the risk and worsens prognosis; CKD is recognised as an important and independent risk factor for CVD [2].

CKD is a serious, debilitating and potentially expensive disease to treat with complications placing a huge burden on healthcare resources [1]. In the later stages of CKD, where there is progression to end-stage renal disease (ESRD), the focus is on management of complications and preparation and implementation of renal replacement therapy [3]. ESRD is serious and its management is costly and unaffordable for many countries. Only a few wealthy countries and individuals with access to private health insurance can meet the demands imposed by the late stages of CKD [1]. Notably, these options are frequently not available to the poor due to high costs nor in developing nations with limited resources and where renal transplantation may not even be available [3]. This leads almost inevitably to premature mortality and impacts hugely on families, both emotionally and economically with loss of income from breadwinners who are usually of working-age.

Thus, there is an urgent need to shift attention in the management of CKD from ESRD to prevention, and early detection and retarding progression to ESRD [1, 3]. Since the earlier stages of CKD may be asymptomatic, screening is necessary for early detection and timely interventions to prevent, delay or reverse subsequent disease progression [3, 4].

To address the burden of CKD, there needs to be an increased awareness and understanding of the problem to encourage regular screening. This can only be achieved by assessing and highlighting the burden of the problem through epidemiological studies of the incidence, prevalence and determinants of CKD [1, 5]. Such data can inform the appropriate allocation of resources to prevent CKD and encourage its early detection to pre-empt progression to ESRD. This is particularly relevant in resource-limited setting such as South Africa. However, in South Africa, there is a dearth of such data with only a few studies reporting on the burden of CKD in general populations [6–8].

Furthermore, there are several formulae to estimate glomerular filtration rate (GFR), the accepted best overall index of renal function [9]. The GFR estimators commonly used are the Cockcroft-Gault formula (CGF) [10], the Modification of Diet in Renal Disease (MDRD) [11] and the recently developed CKD Epidemiology Collaboration (CKD-EPI) equations [9, 12]. These formulae use serum creatinine concentrations, as advocated for screening [7]; however, additional CKD-EPI equations include cystatin C. Cystatin C has been evaluated as an alternative filtration marker to serum creatinine and has been reported to be a better marker than creatinine [12]; estimated GFR (eGFR) based on cystatin C equations may be used as a confirmatory test for renal disease [13]. Few studies in Africa have estimated and compared GFR and determined the prevalence of CKD by these equations simultaneously.

The aim of this study was to determine the prevalence, distribution, concordance and cardiometabolic associations of CKD determined by five eGFR formulae in urban black residents of Cape Town. The eGFR equations used include the CGF [10], the more popular MDRD [11, 14], the creatinine-based CKD-EPI (CKD-EPIcr) equation [9] recommended for the initial assessment of GFR and the two cystatin C-based CKD-EPI equations [12]. The MDRD and CKD-EPI equations have not been adjusted for ethnicity as recommended for African Americans because this has been reported to overestimate kidney function in black South Africans [15]. Similarly, these ethnicity coefficients did not perform well in other Sub-Saharan African populations [16], African Europeans and indigenous Australians [17]. GFR was best estimated when the ethnicity factor was disregarded because the performance of the CKD-EPI and MDRD GFR estimates did not improve with these ethnic adjustments [16, 17].

## Methods

### Study population, sampling procedure and data collection

This cross-sectional study was conducted in men and women residing in the predominantly black townships of Langa, Guguletu, Crossroads, Nyanga and Khayalitsha in Cape Town. Based on an estimated diabetes prevalence of 8% with a precision of 1.5% two-sided with 95% confidence, a sample size of 1000 was planned. Participants were randomly selected in 2008/09 using a 3-stage cluster sampling procedure, which has previously been described in detail [18]. Aerial maps of each township were used to randomly select residential blocks within the main strata followed by selection of individuals from households using quotas. Quotas, calculated using the most recent census, were pre-specified by age and gender categories. Sampling across age groups was disproportionate with older age groups over-sampled to ensure at least 50 men and women in each gender category. Participants who were unable to give consent, bedridden, pregnant or lactating, residents of Cape Town for less than 3 months, on antiretrovirals or tuberculosis treatment or had received cancer treatment within the previous year were ineligible for this study.

Trained fieldworkers conducted the data collection which comprised administered questionnaires, clinical assessments and biochemical analyses. This was performed in accordance with the relevant guidelines and regulations. Fieldworkers administered structured questionnaires to obtain socio-demographic information, self-reported medical history, and data on tobacco (WHO STEPwise surveillance questionnaire) [19] and alcohol use (CAGE set of four questions) [20]. Assets that defined wealth were recorded and included ownership of consumer items (durable goods), access to electricity, and the source of drinking water and toilet facilities.

Clinical assessments comprised anthropometric and blood pressure (BP) measurements. Anthropometric data included height, weight, and waist and hip circumferences and were measured using standardised techniques [21]. Weight was measured to the nearest 0.1 kg using a calibrated scale with the participants barefoot and in light clothing while a stadiometer measured height to the nearest 0.1 cm. Waist and hip circumferences were measured to the nearest 0.1 cm with a flexible tape measure. The tape measure was held parallel to the floor while the waist circumference was measured approximately 2 cm or two finger spacings above the umbilicus. Hip circumference was measured at the maximum posterior protuberance of the buttocks with participants standing upright and their feet together.

After the participant had been seated for 5 min, three BP measurements were taken at two-minute intervals with an Omron BP monitor using an appropriately sized cuff. The average of the second and third BP measurements was used for analysis.

Blood samples, for glucose and lipid estimations, were drawn following a10-hour overnight fast. A standard oral glucose tolerance test with 75 g of anhydrous glucose dissolved in 250 ml of water was thereafter administered, and blood samples drawn 120 min later [22]. Blood samples were kept on ice and transported to the laboratory within 6 h to be centrifuged, aliquoted and stored at − 80° until the assays were performed.

Additional blood specimens were stored at − 80 degrees Celsius and analysed in 2018/2019. The latter biochemical analyses included serum creatinine and cystatin C levels, which were used to determine eGFR. Cystatin C was measured on the Roche 502 platform by a particle enhanced immunoassay which has been standardised against ERM-DA471/IFCC reference material. Serum creatinine was measured by Jaffe kinetic method which is IDMS traceable.

### Definitions

CKD was determined using the CKD-EPIcr eq. (2009) [9], the CKD-EPI cystatin C (CKD-EPIcys) and creatinine–cystatin C (CKD-EPIcr-cys) eqs. (2012) [12], the MDRD (1999) [11] and the CGF (1976) [10]. These equations are complex formulae that include age and gender (in all the above equations) or body mass (CGF only) in various computations together with serum creatinine or serum cystatin C in specific equations. CKD was defined as eGFR of < 60 mL/min/1.73 m^2^. However, this threshold does not distinguish kidney disease from kidney aging and may therefore not be applicable for all ages [23]. Consequently, CKD cut-points were further adjusted for age-specific thresholds as follows: < 40 years: < 75 ml/min per 1.73 m^2^, 40–65 years: < 60 ml/min per 1.73 m^2^, and > 65 years: < 45 ml/min per 1.73 m^2^.

Problematic alcohol use was deemed present if ≥2 of the CAGE set of four questions were answered affirmatively [20]. Smoking ≥1cigarette/day categorised participants who smoked daily. Based on the assets that defined wealth, a principal component analysis of the pooled data was used to develop an asset index [24]. Categories of relative wealth were created using tertiles; the lowest tertile represents the poorest participants.

Body mass index (BMI) was calculated as weight in kilograms divided by height in metres squared (kg/m^2^) and obesity defined as BMI ≥30 kg/m^2^ [25]. Central obesity was computed as waist circumference > 94 cm in men and > 80 cm in women, waist-to-hip ratio as ≥0.9 in men and ≥ 0.85 in women [26] and waist-to-height ratio as > 0.5 [27]. Hypertension was defined as BP ≥140/90 mmHg or the use of antihypertensive agents [28]. Resting heart rate of ≥90 beats per minute was categorised as high.

Diabetes was diagnosed using the 1998 WHO criteria of fasting plasma glucose ≥7.0 mmol/l and/or 2-h post glucose load ≥11.1 mmol/l [22], and included participants with known diabetes. Dyslipidaemia was defined as follows [29]: total cholesterol > 5 mmol/l, triglycerides > 1.5 mmol/l, high-density lipoprotein cholesterol (HDL-C) < 1.2 mmol/l and low-density lipoprotein cholesterol (LDL-C) > 3.0 mmol/l calculated using the Friedewald equation [30] or taking anti-lipid agents. Metabolic syndrome was defined according to the Joint Interim Statement criteria as the presence of any three abnormal findings out of five components [31].

### Statistical analyses

Data analyses were conducted using STATA 15. Univariable analyses (sociodemographic and CVD risk characteristics) are presented as crude mean values and standard deviations for continuous data, and as crude percentages for categorical data. The agreements between the kidney function estimators were determined on a continuous scale using the Pearson correlation tests, and differences between the correlation coefficients were tested. Cohen’s kappa statistic was used to test the agreement of CKD between the various formulae.

Logistic regressions analyses were used to investigate the association of cardiometabolic risk profile with prevalent CKD in age, gender and BMI ≥30 kg/m^2^ adjusted models. Thereafter, each cardiometabolic variable was modelled independently. The data are presented as odds ratios (OR) and 95% confidence intervals (CI). A *p*-value < 0.05 characterised statistically significant findings.

Ethics approval was obtained from the South African Medical Research Council’s Human Research Ethics Committee (EC026–9/2016) and the University of Cape Town’s Research and Ethics Committee (224/2006). All participants signed informed consent.

## Results

Of the 1116 participants examined in this study, 24 were excluded because they did not have creatinine and cystatin C results to determine eGFR. This was mainly because of insufficient stored blood samples. The final study sample included 1092 participants > 21 years of age and comprised 392 men and 700 women.

**Fig. 1:**
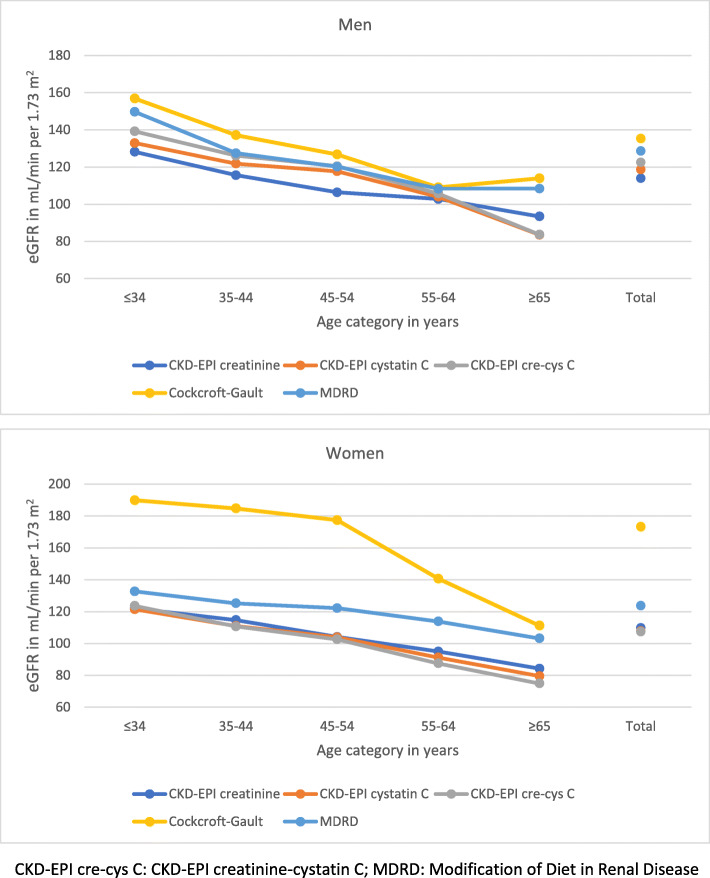
Distribution of mean estimated glomerular filtration rate (eGFR) presented by five formulae in men and women: three CKD-EPI equations, Cockcroft-Gault and MDRD formulae

The mean eGFR was between 114 mL/min/1.73 m^2^ (CKD-EPIcr) and 135 mL/min/1.73 m^2^ (CGF) in men, and 108 mL/min/1.73 m^2^ (CKD-EPIcr-cys) and 173 mL/min/1.73 m^2^ (CGF) in women **(**Fig. 1**)**. Compared with women, mean eGFR in men was significantly higher by the CKD-EPI formulae and lower by CGF (both *p* < 0.05); there was no significant difference by MDRD. By age category, mean eGFR generally decreased significantly with older age in both men and women (*p* < 0.001).

The correlations between different estimates of GFR were positive and significant, and there were no significant differences between any two coefficients. The highest correlation coefficients between the eGFR equations were for CKD-EPIcys and CKD-EPIcr-cys (total sample: 0.994, men: 0.996, women: 0.995), **(**Table 1**)**. eGFR by the cystatin C equations correlated only modestly with CKD-EPIcr.

**Table 1:** Correlation coefficients between the five estimated glomerular filtration rate formulae in urban black men and women

	CKD-EPI creatinine	CKD-EPI cystatin C	CKD-EPIcr-cys	Cockcroft-Gault	MDRD
**Total**					
CKD-EPI creatinine	1	0.460	0.453	0.681	0.814
CKD-EPI cystatin C	0.460	1	0.994	0.341	0.473
CKD-EPIcr-cys	0.453	0.994	1	0.335	0.477
Cockcroft-Gault	0.681	0.341	0.335	1	0.779
MDRD	0.814	0.473	0.477	0.779	1
**Men**					
CKD-EPI creatinine	1	0.368	0.366	0.796	0.863
CKD-EPI cystatin C	0.368	1	0.996	0.407	0.443
CKD-EPIcr-cys	0.366	0.996	1	0.403	0.440
Cockcroft-Gault	0.796	0.407	0.403	1	0.839
MDRD	0.863	0.443	0.440	0.839	1
**Women**					
CKD-EPI creatinine	1	0.525	0.510	0.702	0.790
CKD-EPI cystatin C	0.525	1	0.995	0.403	0.496
CKD-EPIcr-cys	0.510	0.995	1	0.404	0.502
Cockcroft-Gault	0.702	0.403	0.404	1	0.817
MDRD	0.790	0.496	0.502	0.817	1

*CKD-EPIcr-cys* CKD-EPI creatinine-cystatin C, *MDRD* Modification of Diet in Renal Disease

The CKD-EPIcr and MDRD equations were strongly correlated in the total sample (0.814), men (0.863) and women (0.790). MDRD and CGF were also well correlated (total sample: 0.779, men: 0.839, women: 0.817).

**Fig. 2:**
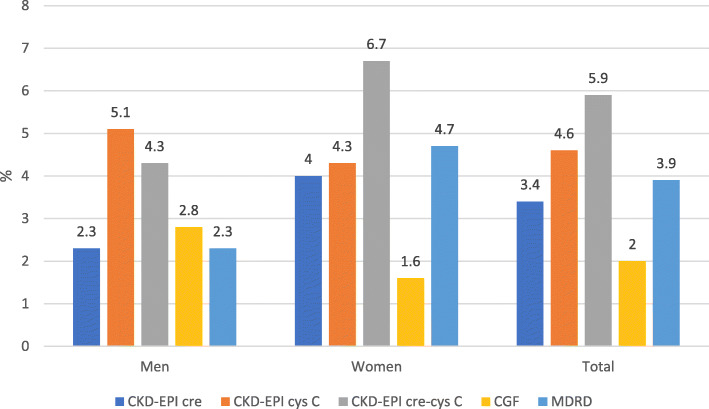
Prevalence (%) of chronic kidney disease (CKD) by three CKD-EPI, Cockcroft-Gault and MDRD formulae in men and women

By the various equations, the prevalence of CKD ranged from 2.0% (CGF) to 5.9% (CKD-EPIcr-cys) in the total sample, 2.3% (CKD-EPIcr and MDRD) to 5.1% (CKD-EPIcys) in men and 1.6% (CGF) to 6.7% (CKD-EPIcr-cys) in women **(**Fig. 2**)**. There was no significant difference in the prevalence of CKD between men and women except by MDRD (*p* = 0.046).

**Fig. 3:**
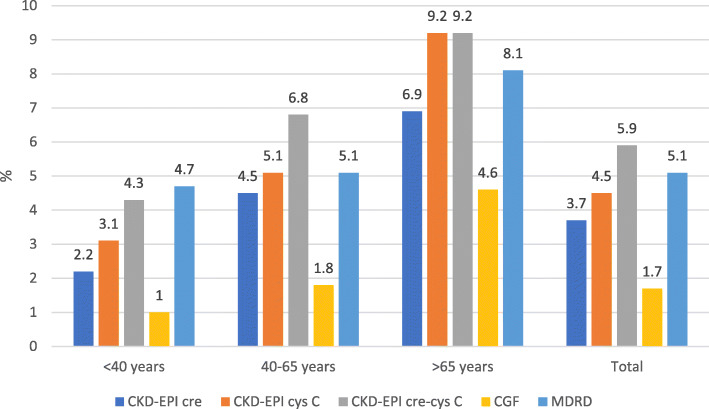
Prevalence (%) of chronic kidney disease (CKD) by three CKD-EPI, Cockcroft-Gault and MDRD formulae using age-specific thresholds

The overall CKD prevalence by age-specific thresholds ranged from 1.7% (CGF) to 5.9% (CKD-EPIcr-cys) **(**Fig. 3**)**. By age groups, CKD prevalence was as follows: < 40 years old:1.0% (CGF) to 4.7% (MDRD), 40–65 years old: 1.8% (CGF) to 6.8% (CKD-EPIcr-cys), and > 65 years old: 4.6% (CGF) to 9.2% (CKD-EPIcys and CKD-EPIcr-cys). CKD prevalence by age-specific thresholds increased with age but was significantly different only for CKD-EPIcr (*p* = 0.043) and for CKD-EPIcys (*p* = 0.027).

The kappa statistic for CKD was high between CKD-EPIcr and MDRD (0.934), and between CKD-EPIcys and CKD-EPIcr-cys (0.815), while the lowest kappa statistic was between CKD-EPIcr-cys and CGF (0.353) **(**Table 2**)**. The kappa statistics for the other comparisons were moderate in agreement (0.410–0.565).

**Table 2:** Cross classification of participants with chronic kidney disease by the various formulae and the kappa statistic for each comparison

	CKD-EPI cystatin C		CKD-EPIcr-cys		Cockcroft-Gault		MDRD	
	yes	no	yes	no	yes	no	yes	no
CKD-EPI creatinine								
**yes**	21	16	22	15	17	20	37	0
**no**	29	1026	42	1013	5	1050	5	1050
**Kappa**	0.462		0.410		0.565		0.934	
CKD-EPI cystatin C								
**yes**			47	3	16	34	21	29
**no**			17	1025	6	1036	21	1021
**Kappa**			0.815		0.429		0.433	
CKD-EPIcr-cys								
**yes**					16	48	23	41
**no**					6	1022	19	1009
**Kappa**					0.353		0.406	
Cockcroft-Gault								
**yes**							17	5
**no**							25	1045
**Kappa**							0.519	

Number of participants with chronic kidney disease (CKD): CKD-EPI creatinine: *n* = 37; CKD-EPI cystatin C: *n* = 50; CKD-EPI creatinine-cystatin C (CKD-EPIcr-cys): *n* = 64; Cockcroft-Gault: *n* = 22; Modification of Diet in Renal Disease (MDRD): *n* = 42

The prevalence of participants who had eGFR of 60–90 mL/min/1.73 m^2^ by the creatinine-defined formulae but CKD on CKD-EPIcys and CKD-EPIcr-cys is as follows: CKD-EPIcr (*n* = 142): 12.7 and 16.2%, CGF (*n* = 120): 13.3 and 16.7%, and MDRD (*n* = 221): 9.1 and 11.3%, respectively (Supplementary Figure 1).

The prevalence of CKD by the CKD-EPIcr was significantly higher in participants who were older, pensioners, lived in formal housing and had a history of heart attack or stroke (Supplementary Table 1). Participants with compared to without cardiometabolic diseases generally had significantly higher prevalence of CKD across the eGFR formulae (Supplementary Figure 2). However, the CKD prevalence was not significantly higher for LDL-C determined by CKD-EPIcys and CGF, and for metabolic syndrome determined by CGF in participants with compared to without these conditions.

Measures of adiposity (except for waist-to-hip ratio), systolic and diastolic BPs and hypertension, fasting and 2-h glucose and diabetes, and metabolic syndrome were significantly higher in participants with compared to without CKD **(Supplementary Table** **2****)**. Mean lipid levels and dyslipidaemia, except for mean HDL-C, and prevalence of low HDL-C and raised triglycerides, were significantly higher in participants with compared to without CKD.

In the age, gender and BMI adjusted regression model (basic model), ages 55–64 years and ≥ 65 years and BMI ≥30 kg/m^2^ were significantly associated with CKD-EPIcr-defined CKD **(**Table 3**)**. When the cardiometabolic variables were entered separately and individually in the above model, hypertension (OR: 2.57, 95% CI: 1.01–6.50), heart rate ≥ 90 beats/min (OR: 3.98, 95%CI: 1.56–10.14), diabetes (OR: 2.09, 95% CI: 1.01–4.30) and increasing LDL-C (OR: 1.44, 95% CI: 1.02–2.02) were significant. The model with metabolic syndrome, adjusted for age and gender but not BMI ≥30 kg/m^2^, was also significant (OR: 2.70, 95% CI: 1.20–6.06).

**Table 3:** Multiple logistic regression models (odds ratios and 95% confidence intervals) for the associations with chronic kidney disease (CKD) determined by the CKD-EPI, Cockcroft-Gault and MDRD formulae

	CKD-EPI creatinine	CKD-EPI cystatin C	CKD-EPI creatinine-cystatin C	Cockcroft-Gault	MDRD
Age in years: < 34	1.00	1.00	1.00	1.00	1.00
35–44	0.89 (0.15–5.40)	7.99 (0.95–66.87)	1.66 (0.50–5.50)	2.74 (0.25–30.26)	1.38 (0.34–5.60)
45–54	3.76 (1.00–14.17)	**12.92 (1.64–102.02)**	**3.50 (1.22–10.01)**	6.20 (0.68–56.27)	**3.65 (1.14–11.71)**
55–64	**10.15 (2.80–36.82)**	**29.05 (3.73–226.48)**	**9.83 (3.56–27.17)**	**10.25 (1.13–92.96)**	**7.90 (2.49–25.11)**
≥ 65	**14.95 (4.03–55.52)**	**98.09 (12.92–745.01)**	**22.20 (8.05–61.24)**	**53.55 (6.74–425.36)**	**11.81 (3.62–38.49)**
Gender: female	1.13 (0.47–2.73)	0.53 (0.26–1.09)	1.23 (0.63–2.41)	0.61 (0.23–1.65)	1.56 (0.66–3.64)
BMI ≥30 kg/m^2^	**2.81 (1.21–6.55)**	**2.60 (1.26–5.38)**	**1.98 (1.06–3.69)**	0.81 (0.29–2.24)	2.04 (0.95–4.35)
Hypertension	**2.57 (1.01–6.50)**	1.73 (0.83–3.58)	1.70 (0.89–3.24)	3.09 (0.95–10.04)	**2.61 (1.13–6.04)**
Heart rate ≥ 90 beats/min	**3.98 (1.56–10.14)**	1.96 (0.70–5.53)	1.54 (0.60–3.95)	1.91 (0.41–8.98)	**3.14 (1.27–7.74)**
Diabetes	**2.09(1.01–4.30)**	1.30 (0.66–2.57)	1.02 (0.54–1.93)	1.81 (0.69–4.73)	1.82 (0.91–3.66)
Increasing TC	1.15 (0.86–1.54)	1.06 (0.81–1.38)	1.04 (0.82–1.31)	1.14 (0.78–1.65)	1.15 (0.87–1.50)
Increasing HDL-C	0.45 (0.15–1.35)	0.79 (0.36–1.73)	0.65 (0.31–1.38)	0.60 (0.19–1.88)	0.54 (0.21–1.42)
Increasing triglycerides	1.23(0.99–1.54)	1.17 (0.94–1.45)	1.10 (0.87–1.40)	1.21 (0.96–1.54)	1.20 (0.98–1.47)
Increasing LDL-C	**1.44(1.02–2.02)**	1.18 (0.87–1.62)	1.18 (0.90–1.56)	1.52 (0.98–2.33)	**1.39 (1.02–1.91)**
Increasing HDL-C: TC ratio	0.96 (0.90–1.01)	1.01 (0.99–1.04)	1.00 (0.98–1.03)	0.98 (0.93–1.03)	0.96 (0.91–1.01)
Metabolic syndrome	**2.70 (1.20–6.06)**	1.16 (0.61–2.22)	1.15 (0.65–2.06)	1.38 (0.54–3.57)	**2.99 (1.39–6.46)**

Significant values (*p* < 0.05) are in bold. MDRD: Modification of Diet in Renal Disease, BMI: body mass index; TC: total cholesterol; HDL-C: high-density lipoprotein cholesterol; LDL-C: low-density lipoprotein cholesterol. The models with metabolic syndrome were adjusted for age and gender but not BMI ≥30 kg/m^2^. When the cardiometabolic variables were entered separately and individually in the basic model (age, gender and body mass index) for CKD determined by the CDK-EPI creatinine formula, there was no change in the direction or significance of the other variables except for the model with heart rate where age 45–54 years was now significant. For CKD determined by CKD-EPI cystatin C, CKD-EPI creatinine/cystatin C and MDRD formula, age 45–54 years and BMI ≥30 kg/m^2^ were no longer significant in some models when the cardiometabolic variables were entered separately and individually in the basic models. Age 55–64 years was not significant in the Cockcroft-Gault models with hypertension, LDL-C or metabolic syndrome

In the basic models for CKD determined by the other eGFR equations, older age was generally significant while BMI ≥30 kg/m^2^ was significant for the CKD-EPI equations **(**Table 3**)**. Cardiometabolic variables were not significant when entered separately and individually in these models except for MDRD derived CKD. Hypertension, raised heart rate, increasing LDL-C and metabolic syndrome were significantly associated with MDRD derived CKD.

## Discussion

To our knowledge, this study is among the first to examine and compare eGFR and the prevalence of CKD derived from multiple formulae, including the cystatin C-based equations, across age and gender categories in an urban black community in South Africa and Sub-Saharan Africa in general. The prevalence of CKD, derived from the three CKD-EPI equations, CGF and MDRD, ranged from 2% (CGF) to 6% (CKD-EPIcr-cys) in the total sample. This was similar to a recent systematic review and meta-analysis of CKD prevalence studies from Africa (4.6%) [32], but slightly lower than the MDRD-defined CKD prevalence of 6.3% in black South Africans from Soweto, Johannesburg [7]. Compared with this study, CKD prevalence in Cape Town was also higher in a mixed ancestry population (CKD-EPIcr: 17.3%; MDRD: 23.9%) [6] and among teachers (MDRD or proteinuria: 10.4%) [8]. The higher CKD prevalence in the latter study may be attributed to the inclusion of proteinuria in the diagnosis, and in the former to the mean older age (53 years) and higher prevalence of diabetes (26.4%) compared with this sample; hypertension prevalence was not reported.

CKD prevalence was higher by the cystatin C equations compared with the creatinine-based equations including CKD-EPIcr. A large proportion of participants identified with CKD by the cystatin C-based equations did not have CKD by the creatinine-based equations. Cystatin C-based equations compared with creatinine-based formulae have been suggested to more precisely estimate GFR in black South Africans with early renal disease [15]. Apart from GFR, cystatin C levels are not affected by many factors that influence serum creatinine such as muscle mass, diet, gender and age.

Additionally, the two cystatin C based equations were fair-to-moderately correlated with the CKD-EPIcr, MDRD and CGF. This suggests that the current creatinine-based equations used to determine CKD in the black South African population in clinical practice may perhaps not be the best estimators if the cystatin C equations are considered superior to the creatinine-based formulae. The cystatin C equations have been reported to show better precision and accuracy in determining GFR compared with CKD-EPIcr; however, this is mainly in western populations [33]**.**

Bukabau and colleagues report that in a study of 494 adults from two Sub-Saharan African countries equations with cystatin C alone or in combination with creatinine did not improve GFR estimates or add substantial value over CKD-EPIcr when compared with measured GFR [16]. However, a Congolese study of 93 healthy adults found that CKD-EPIcys performed better than CKD-EPIcr when compared with measured GFR [34]. Nevertheless, the authors reported that slightly better performance of the former equation was not enough to warrant the additional costs of the cystatin C marker in daily practice. The cost of performing the cystatin C assay is high and there are delays in turnaround time because of the need for analyses to be conducted in batches [15] making the frequent utilisation of this test problematic. Therefore, concerns remain about the cost-effectiveness and clinical significance of using cystatin C-based eGFR estimators over creatinine-based formulae in general populations [33]. It may therefore be pragmatic to assess cystatin C if creatinine-based eGFR is 60–74 mL/min per 1.73 m^2^ without albuminuria to obtain a better estimate of GFR [12]. However, the 2013 Kidney Disease Improving Global Outcomes (KDIGO) CKD Guideline Development Work Group suggests measuring cystatin C for confirmation of CKD only when markers of kidney damage are absent and eGFR by creatinine-based formulae is 45–59 mL/min per 1.73 m^2^ [13]. Further research is necessary to establish guidelines in the local population.

The differences in CKD prevalence estimates between the creatinine and cystatin C based equations highlights the need for South African population-based studies with adequate sample sizes to validate these eGFR formulae. This will provide the evidence needed to determine the appropriate eGFR equation to use in the local setting. Further, if the latter is found to include cystatin C, it may provide an impetus for improving cystatin C assays and decreasing costs to enable widespread utility of the test.

However, with regards to significant associations between CKD and cardiometabolic diseases, these were best demonstrated with CKD-EPIcr followed by MDRD in the adjusted models. There were no significant associations between any cardiometabolic parameter tested and CKD determined by either cystatin C-based CKD formulae when adjusted for age, gender and obesity. This further underscore the need for in-depth research on CKD in the local population.

The similar findings of significant associations in the adjusted models for CKD-EPIcr and MDRD, as well as their good correlations, is not surprising. This is because both formulae are based on the same four variables although the forms of the variables and the coefficients differ. The strong agreement between CKD-EPIcr and MDRD was also reported in the mixed ancestry South African population [6].

The overall prevalence of CKD by a single threshold value compared with age-specific thresholds was similar. However, the age-specific thresholds identified different individuals with CKD compared with those identified using a single threshold. The age-specific vs. the single threshold cut-point identified a higher proportion of younger participants < 40 years old (1.0–4.7% vs. 0.4–1.4%, respectively) and a lower proportion of older participants over 65 years of age (4.6–9.2% vs. 12.6–25.3%, respectively). Therefore, the single threshold of 60 ml/min per 1.73 m^2^ may overlook CKD in younger individuals who do not display overt signs of kidney damage; notably, mortality in individuals < 40 years of age has been reported to increase at GFR < 75 ml/min per 1.73 m^2^ [23]. Therefore, it is important to optimally identify younger adults with early onset CKD to prevent progressive kidney damage and adverse health outcomes.

In contrast, in > 65-year-old adults, the risk of mortality increases only at GFR < 45 ml/min per 1.73 m^2^ [23]. Therefore, the threshold of 60 ml/min per 1.73 m^2^ may over-diagnose CKD in the elderly; a large proportion of older adults with eGFR between 45 and 59 ml/min per 1.73 m^2^ and no other signs of kidney damage should perhaps not be classified as having CKD. Incorrect classification and diagnosis can lead to inappropriate care and its associated adverse impacts such as unnecessary healthcare expenditure and undue stress, among other factors. Using age-specific CKD thresholds may improve resource allocation with care directed to those at higher risk of CKD associated adverse outcomes [23].The higher prevalence of CKD in participants with compared to without cardiometabolic disease risk factors, particularly hypertension and diabetes, reflect their key roles in renal damage. Reinforcing this, was that hypertension, high heart rate, increasing LDL-C and metabolic syndrome were individually and separately related to CKD by CKD-EPIcr and MDRD in the adjusted models while diabetes was associated with CKD-EPIcr only. This highlights the need for regular screening of individuals with cardiometabolic diseases to identify CKD as well as for optimal management of hypertension and diabetes to prevent renal and other complications [2].

### Strengths and limitations

The strength of this study includes the large sample size with a wide age range that included both sexes. Another strength is the determination of eGFR using five equations including cystatin C. The findings of this study may be generalised to urban black populations in other South African cities with similar age demographics, cardiometabolic patterns and other renal-related disorders.

A major limitation is the absence of measured GFR to compare the estimated GFR values against; hence, eGFR could not be validated against a gold standard. This would have identified the eGFR equation that best correlated with measured GFR and which could be use in this population. It would have been ideal to repeat testing after 3 months to confirm a diagnosis of CKD for accurate prevalence rates; however, this was not possible and is a drawback of this study. Neither was albuminuria nor other markers of renal damage determined to more accurately diagnose CKD. CKD classification includes the presence of albuminuria even if eGFR is ≥60 mL/min/1.73 m^2^; therefore, we could have underestimated the prevalence in this study. Our study could have been underpowered to reliably estimate the prevalence of CKD. A larger sample size may have been required to estimate CKD prevalence (2–7%) compared with that required for an 8% diabetes prevalence. The cross-sectional design of this study precluded conclusions on causality between cardiometabolic diseases and CKD. A further limitation, and a common characteristic of epidemiological studies in South Africa, is the low sample realisation in men (64%), which may be related to their unwillingness to participate, particularly for the drawing of blood samples.

## Conclusions

CVDs and other non-communicable diseases are drawing increasing attention in South Africa; CKD, a key non-communicable disease and CVD risk factor should not be overlooked because of the potential public health implications. Screening for CKD should be incorporated into the routine assessment of high-risk patients such as those with hypertension or diabetes. However, this study highlights that CKD prevalence estimates can vary within a population because of the different eGFR equations used. This underscores the need to improve the accuracy of CKD diagnoses. Further research is needed in the local setting to determine and validate the appropriate measure to use when estimating renal function. Ongoing CKD surveillance to monitor the burden is equally important and should be considered in resource limited settings like South Africa to pre-empt future escalations in the CKD burden.

## Supplementary information

**Additional file 1: Figure S1.** Number of participants with estimated glomerular filtration rate (eGFR) of 60–90 mL/min per 1·73 m^2^ by the creatinine-based formulae that have chronic kidney disease by the cystatin C-based formulae.

**Additional file 2: Table S1.** Socio-demographic characteristics, lifestyle behaviours and medical history presented by chronic kidney disease status determined by the CKD-EPI creatinine formula.

**Additional file 3: Figure S2.** Prevalence of chronic kidney disease (CKD) by five eGFR formulae in participants with and without selected cardiometabolic diseases.

**Additional file 4: Table S2.** Cardiometabolic variables presented by chronic kidney disease status determined by the CKD-EPI creatinine formula.

## Abbreviations

BMI: Body mass index
BP: Blood pressure
CGF: Cockcroft-Gault formula
CKD: Chronic kidney disease
CKD-EPI: CKD Epidemiology Collaboration
CKD-EPIcr: CKD-EPI creatinine
CKD-EPIcr-cys: CKD-EPI creatinine-cystatin C
CKD-EPIcys: CKD-EPI cystatin C
CVD: Cardiovascular disease
eGFR: Estimated glomerular filtration rate
ESRD: End-stage renal disease
GFR: Glomerular filtration rate
HDL-C: High-density lipoprotein cholesterol
LDL-C: Low-density lipoprotein cholestero
MDRD: Modification of Diet in Renal Disease

## Availability of data and materials

The datasets generated during and/or analysed during the current study are not publicly available because further analyses are currently being conducted by the investigators but are available from the corresponding author on reasonable request.
