# Supplementary material for: Prevalence, concordance and associations of chronic kidney disease by five estimators in South Africa
Source: BMC Nephrol. 2020 Aug 27;21:372. doi: 10.1186/s12882-020-02018-x (PMC7451105; doi:10.1186/s12882-020-02018-x)
Supplement: Supplementary file 1 — Additional file 1: Figure S1. Number of participants with estimated glomerular filtration rate (eGFR) of 60–90 mL/min per 1·73 m2 by the creatinine-based formulae that have chronic kidney disease by the cystatin C-based formulae. [file 12882_2020_2018_MOESM1_ESM.docx]

**Supplementary Figure 1: Number of participants with estimated glomerular filtration rate (eGFR) of 60-90 mL/min per 1·73 m² by the creatinine-based formulae that have chronic kidney disease by the cystatin C-based formulae**
