# Supplementary material for: Prevalence, concordance and associations of chronic kidney disease by five estimators in South Africa
Source: BMC Nephrol. 2020 Aug 27;21:372. doi: 10.1186/s12882-020-02018-x (PMC7451105; doi:10.1186/s12882-020-02018-x)
Supplement: Supplementary file 2 — Additional file 2: Table S1. Socio-demographic characteristics, lifestyle behaviours and medical history presented by chronic kidney disease status determined by the CKD-EPI creatinine formula. [file 12882_2020_2018_MOESM2_ESM.docx]

**Supplementary Table 1: Socio-demographic characteristics, lifestyle behaviours and medical history presented by chronic kidney disease status determined by the CKD-EPI creatinine formula**

| **Glomerular filtration rate, mL/min per 1·73 m²** | **Total** | **<60** | **≥60** | **p-value** |
| --- | --- | --- | --- | --- |
| Number | 1092 | 37 | 1055 |  |
| **Socio-demographic characteristics** |  |  |  |  |
| Age in years, mean ±SD | 43.5 ±13.2 | 57.0 ±12.3 | 43.0 ±13.0 | **<0.001** |
| Gender, %: |  |  |  | 0.135 |
| Female | 64.1 | 75.7 | 63.7 |  |
| Male | 35.9 | 24.3 | 36.3 |  |
| % of life spent in urban area, mean ±SD (years) | 61.7 ±32.6 | 70.4 ±27.7 | 61.4 ±32.7 | 0.098 |
| Education, %: |  |  |  | 0.183 |
| <7 years | 38.2 | 48.7 | 37.8 |  |
| ≥7 years | 61.8 | 51.4 | 62.2 |  |
| Employment Status, %: |  |  |  | **<0.001** |
| Employed | 21.3 | 13.5 | 21.6 |  |
| Unemployed | 58.2 | 35.1 | 59.1 |  |
| Pensioners | 14.7 | 40.5 | 13.7 |  |
| Other* | 5.8 | 10.8 | 5.6 |  |
| Housing type, %: |  |  |  | **0.006** |
| Informal shack | 43.9 | 21.6 | 44.6 |  |
| Built formal units, council/core house/hostel | 56.1 | 78.4 | 55.4 |  |
| Wealth tertiles, %: |  |  |  | 0.331 |
| 1st (poorest) | 32.9 | 21.6 | 33.3 |  |
| 2nd | 33.1 | 37.8 | 32.9 |  |
| 3rd (least poor) | 34.1 | 40.5 | 33.8 |  |
|  |  |  |  |  |
| **Lifestyle behaviours, %** |  |  |  |  |
| Problematic alcohol use: CAGE ≥2 | 28.8 | 8.1 | 29.5 | **0.001** |
| Smoke: ≥1cigarette/day | 22.0 | 0.0 | 22.8 | **0.005** |
|  |  |  |  |  |
| **Self-reported medical history, %** |  |  |  |  |
| Heart attack | 3.9 | 13.5 | 3.5 | **0.002** |
| Stroke | 3.7 | 13.5 | 3.3 | **0.001** |
|  |  |  |  |  |

*Other: comprised homemakers, students and those receiving disability grants; significant p-values are in bold
