# Supplementary material for: Prevalence, concordance and associations of chronic kidney disease by five estimators in South Africa
Source: BMC Nephrol. 2020 Aug 27;21:372. doi: 10.1186/s12882-020-02018-x (PMC7451105; doi:10.1186/s12882-020-02018-x)
Supplement: Supplementary file 3 — Additional file 3: Figure S2. Prevalence of chronic kidney disease (CKD) by five eGFR formulae in participants with and without selected cardiometabolic diseases. [file 12882_2020_2018_MOESM3_ESM.docx]

**Supplementary Figure 2:** **Prevalence of chronic kidney disease (CKD) by five eGFR formulae in participants with and without selected cardiometabolic diseases**

CKD prevalence was significantly higher (p <0.05) in participants with compared to without cardiometabolic diseases except for LDL-C determined by CKD-EPI cystatin C and Cockcroft-Gault (CGF) formulae, and for metabolic syndrome determined by CGF.
