# Supplementary material for: Prevalence, concordance and associations of chronic kidney disease by five estimators in South Africa
Source: BMC Nephrol. 2020 Aug 27;21:372. doi: 10.1186/s12882-020-02018-x (PMC7451105; doi:10.1186/s12882-020-02018-x)
Supplement: Supplementary file 4 — Additional file 4: Table S2. Cardiometabolic variables presented by chronic kidney disease status determined by the CKD-EPI creatinine formula. [file 12882_2020_2018_MOESM4_ESM.docx]

**Supplementary Table 2: Cardiometabolic variables presented by chronic kidney disease status determined by the CKD-EPI creatinine formula**

| **Glomerular filtration rate, mL/min per 1·73 m²** | **Total** | **<60** | **≥60** | **p-value** |
| --- | --- | --- | --- | --- |
| Number | 1092 | 37 | 1055 |  |
| Mean ±SD |  |  |  |  |
| Adiposity: |  |  |  |  |
| Body mass index (kg/m^2^) | 29.8 ±8.4 | 34.2 ±8.7 | 29.6 ±8.3 | **0.001** |
| Waist circumference (cm) | 93.0 ±15.6 | 98.9 ±13.4 | 92.8 ±15.6 | **0.021** |
| Waist-to-hip ratio | 0.86 ±0.1 | 0.88 ±0.1 | 0.86 ±0.1 | 0.346 |
| Waist-to-height ratio | 0.58 ±0.1 | 0.63 ±0.1 | 0.57 ±0.1 | **0.001** |
| Blood pressure (mmHg): |  |  |  |  |
| Systolic | 126.4 ±23.4 | 139.4 ±35.4 | 125.9 ±22.7 | **0.001** |
| Diastolic | 82.0 ±13.2 | 86.3 ±18.2 | 81.9 ±13.0 | **0.044** |
| Heart rate (beats/minute) | 70.3 ±12.4 | 72.8 ±13.6 | 70.2 ±12.3 | 0.216 |
| Glucose (mmol/l): |  |  |  |  |
| Fasting | 5.4 ±2.5 | 6.5 ±3.9 | 5.4 ±2.5 | **0.006** |
| 2-hour | 6. 9±4.2 | 9.0 ±6.7 | 6.9 ±4.1 | **0.004** |
| Lipids: |  |  |  |  |
| Total cholesterol (TC) (mmol/l) | 4.4 ±1.1 | 4.9 ±1.5 | 4.4 ±1.1 | **0.009** |
| High-density lipoprotein cholesterol (HDL-C) (mmol/l) | 1.2 ±0.5 | 1.1 ±0.3 | 1.2 ±0.5 | 0.226 |
| Triglycerides (mmol/l) | 1.1 ±0.9 | 1.6 ±1.4 | 1.1 ±0.9 | **0.005** |
| Low-density lipoprotein cholesterol (LDL-C) (mmol/l) | 3.0 ±1.0 | 3.7 ±1.1 | 3.0 ±0.9 | **<0.001** |
| HDL-C:TC ratio, % | 27.3 ±9.9 | 23.3 ±8.1 | 27.4 ±10.0 | **0.015** |
|  |  |  |  |  |
| Prevalence, % |  |  |  |  |
| Adiposity: |  |  |  |  |
| BMI ≥30 kg/m^2^ | 44.6 | 73.0 | 43.6 | **<0.001** |
| Waist circumference: men >94 cm, women >80 cm | 63.9 | 83.8 | 63.2 | **0.010** |
| Waist-to-hip ratio: men ≥0.9, women ≥0.85 | 45.8 | 54.1 | 45.5 | 0.305 |
| Waist-to-height ratio >0.5 | 70.6 | 86.5 | 70.1 | **0.031** |
| Hypertension | 42.1 | 81.1 | 40.8 | **<0.001** |
| Heart rate ≥90 beats/minute | 6.7 | 18.9 | 6.3 | **0.002** |
| Diabetes | 14.7 | 40.5 | 13.7 | **<0.001** |
| Dyslipidaemia: |  |  |  |  |
| TC >5 mmol/l | 26.5 | 40.5 | 26.0 | **0.048** |
| HDL-C <1.2 mmol/l | 62.7 | 70.3 | 62.5 | 0.334 |
| Triglycerides >1.5 mmol/l | 16.3 | 27.0 | 15.9 | 0.072 |
| LDL-C >3 mmol/l | 47.8 | 64.9 | 47.2 | **0.035** |
| HDL-C:TC ratio <20% | 19.4 | 37.8 | 18.8 | **0.004** |
| Metabolic syndrome | 36.2 | 73.0 | 34.9 | **<0.001** |
|  |  |  |  |  |

Hypertension: BP ≥140/90 mmHg or on hypertension treatment; Diabetes: fasting glucose ≥7.0 mmol/l, 2-hr glucose ≥11.1 mmol/l or known diabetes; significant p-values are in bold
